# Single-cell RNA Sequencing Analysis Reveals New Immune Disorder Complexities in Hypersplenism

**DOI:** 10.3389/fimmu.2022.921900

**Published:** 2022-07-05

**Authors:** Hai-chao Zhao, Chang-zhou Chen, Huang-qin Song, Xiao-xiao Wang, Lei Zhang, Hao-liang Zhao, Jie-feng He

**Affiliations:** ^1^ The Third Hospital of Shanxi Medical University, Shanxi Bethune Hospital, Shanxi Academy of Medical Sciences, Tongji Shanxi Hospital, Taiyuan, China; ^2^ Department of Hepatobiliary Surgery, Shanxi Bethune Hospital, Shanxi Academy of Medical Sciences, Tongji Shanxi Hospital, Taiyuan, China; ^3^ Department of Liver Surgery and Transplantation, Liver Cancer Institute, Zhongshan Hospital, Fudan University, Shanghai, China; ^4^ Key Laboratory of Carcinogenesis and Cancer Invasion (Fudan University), Ministry of Education, Shanghai, China; ^5^ Institute of Biomedical Sciences, Fudan University, Shanghai, China; ^6^ Hepatic Surgery Center, Institute of Hepato-Pancreato-Biliary Surgery, Tongji Hospital, Tongji Medical College, Huazhong University of Science and Technology, Wuhan, China

**Keywords:** hypersplenism, single-cell RNA sequencing, T-cells, B-cells, immune disorder

## Abstract

Hypersplenism (HS) is a concomitant symptom of liver or blood disease. Not only does the treatment of HS face challenges, but the transcriptome of individual cells is also unknown. Here, the transcriptional profiles of 43,037 cells from four HS tissues and one control tissue were generated by the single-cell RNA sequencing and nine major cell types, including T-cells, B-cells, NK cells, hematopoietic stem cells, neutrophil cells, mast cells, endothelial cells, erythrocytes, and dendritic cells were identified. Strikingly, the main features were the lack of *CCL5* ^+^ B-cells in HS and the presence of *SESN1* ^+^ B cells in HS with hepatocellular carcinoma (HS-HCC). In cell-cell interaction analysis, CD74-COPA and CD94-HLA-E in HS were found to be up-regulated. We further explored HS-specifically enriched genes (such as *FKBP5*, *ADAR*, and *RPS4Y1*) and found that *FKBP5* was highly expressed in HCC-HS, leading to immunosuppression. Taken together, this research provides new insights into the genetic characteristics of HS *via* comprehensive single-cell transcriptome analysis.

## Introduction

Hypersplenism (HS), as a clinical syndrome, is mainly characterized by splenomegaly and a significant reduction in one or more blood cells (1, 2). The etiologies of HS are diverse (3, 4). Orrily et al. surveyed 170 patients and reported the main causes of HS, including liver disease (36%), blood disease (35%), infectious disease (6%), inflammatory disease (5%), primary splenic disease (4%), and other (unknown) causes (3%) (5). In Southeast Asian countries, hepatitis cirrhosis and portal hypertension are the most common causes of HS (4). Many treatments for HS, such as splenectomy, partial splenic embolization, trans-jugular intrahepatic portosystemic shunt, and liver transplantation have been applied, but none is entirely satisfactory (1, 6–10). Splenectomy is the mainstay of treatment for HS (11, 12), but it may cause potential symptoms such as post-operative portal vein thrombosis and overwhelming sepsis (7). In addition, patients with significant thrombocytopenia or leukopenia also cannot tolerate interferon treatment for viral hepatitis (1, 7). For liver cancer patients with HS, splenectomy in addition to tumor therapy can improve the immune function of patients and prolong their overall survival (13–15). Some scholars believed that the effect of immunosuppressive cells is alleviated after removal of the spleen (16), but the specific mechanism of action thereof is unknown. Therefore, it is important to reveal more methods of HS, cytokines and growth factor treatments may offer hope in this situation. Therefore, it is necessary to perform studies on characterizing cellular states and molecular circuitries for spleen tissue of patients with HS (1).

Recently, single-cell RNA sequencing (scRNA-seq) has offered new possibilities to address biological and medical questions (17–19). Unlike bulk RNA sequencing, scRNA-seq allows researchers to perform transcriptome analysis at the single-cell level. scRNA-seq has been widely used in research, including cancer disease, developmental biology, immunology, and neuroscience (20, 21). One tumor includes several cancer cell populations, while scRNA-seq method can reveal the inter-tumor and intra-tumor heterogeneity, hepatocellular carcinoma (22), and lung adenocarcinoma (23). Besides that, scRNA-seq was also applied in immunological research to reveal heterogeneity among immune cells and identify novel immune cell populations (24). Together, scRNA-seq has provided insights into different disease that will be beneficial in diseases prevention and therapy.

No investigation using scRNA-seq on HS has yet been reported. The cell types and specific genes in HS are unknown, making it necessary to understand the genetic characteristics of HS and find possible treatments. Hence, in this study, five spleen samples were collected and scRNA-seq was performed through the 10× Genomics sequencing platform. The cell subtypes in each sample and the specific genes for each enriched cell group were investigated. Additionally, the evolutionary trajectory among different types of HS was also constructed to expound the relationship among these HS.

## Results

### ScRNA-Seq of Major Changes in Transcriptional Profiles Between HS Patients and Healthy Controls

Spleen tissues from HS patients with hepatitis B virus (HS-HBV, PH024), portal hypertension (HS-PHT, PH026), hepatitis C virus (HS-HCV, PH027), and hepatocellular carcinoma (HS-HCC, PH037), and one patient with a normal spleen (PN021) were used (**Table 1**). The average age of patients was 54.4 years (42-67 years), and two were male and three were female. All patients with HS showed splenomegaly on imaging. HS patients demonstrated a decrease in blood cells (single or simultaneous reduction of red blood cells, white blood cells, or platelets). After splenectomy, blood cells returned to a near-normal level.

**Table 1:** Clinical characteristics of HCC patients profiled by scRNA-seq and validated by staining in this study, Related to **Figure 1**.

Number	Gender	Age (y/o)	Infectious disease	Red blood cell count (*10^12^)	Hemoglobin (g/L)	White blood cell count (*10^9^)	Platelet count (*10^9^)	Albumin (g/L)
PN021	Male	51	no	3.19	102	10.8	137	28.7
PH024	Male	67	HBV	3.14	86	3.1	30	36.7
PH026	Female	42	no	3.68	109	2.0	35	26.2
PH027	Female	63	HCV	3.60	114	9.9	122	33.1
PH037	Female	49	no	3.40	98	2.3	25	29.9

**Figure 1:**
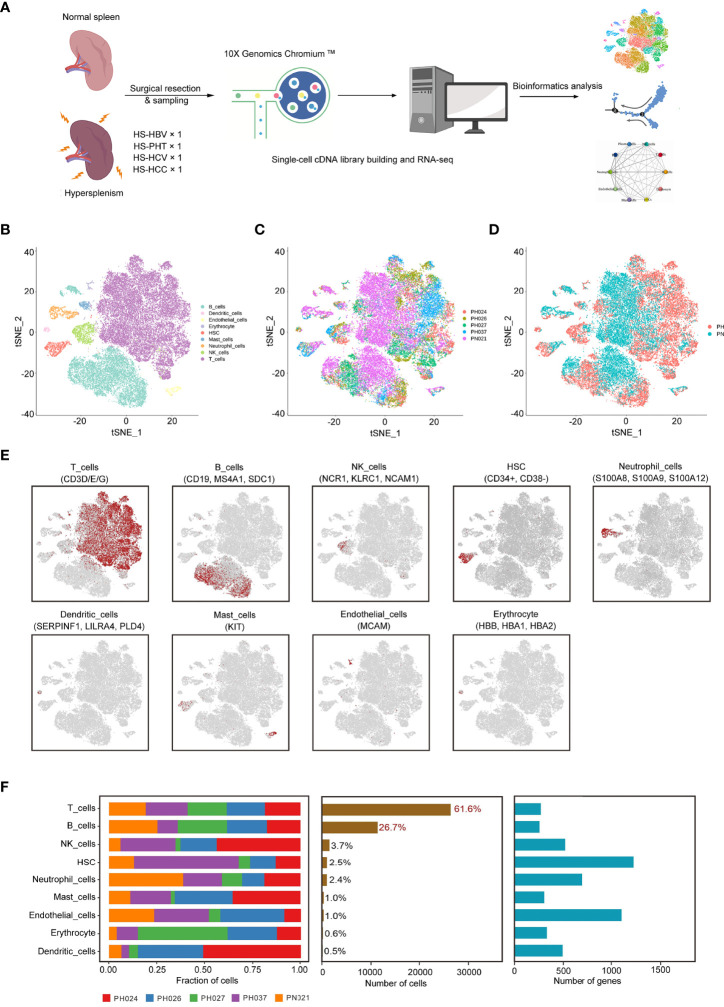
Identification of cell clusters in HS samples with scRNA-seq. **(A)** Schematic representation showing the collection and processing of fresh surgical resection specimens from four HS and one normal tissue for scRNA-seq. **(B)** *t*-SNE plot of the 43,037 cells illustrating nine cell clusters, with each cluster color coded to indicate the associated cell types. **(C)** t-SNE plot of the cell distribution in different HS samples. **(D)** t-SNE plot of the cell distribution in health control and HS group. **(E)** t-SNE plot of the cell cluster, color coding for the expression of the marker genes (gray to red) for the indicated cell subtype. **(F)** Data of the nine clusters from five samples (from left to right): the fraction of cells originating from each patient, the number of cells, and box plots of the number of UMIs and genes.

All samples were digested to a single-cell suspension and studied by 10× Genomics scRNA-seq techniques (**Figure 1A**). After quality control, a total of 43,037 cells were obtained from all samples, of which 24,387 cells (56.7%) were derived from HS patients, about 6,096 cells per HS sample, and 18,650 cells (43.3%) from the normal control sample. The number of cells of control sample was about three times that of the HS sample, and the PH024 and PH037 samples had the least cells. Using t-distributed stochastic neighbor embedding (t-SNE) analysis, all cells were classified in 24 clusters (**Figures S1A–C**), and they were further classified to 9 major cell clusters based on marker genes, including T-cells (61.64%), B-cells (26.72), NK cells (3.68%), hematopoietic stem cells (HSC, 2.47%), neutrophil cells (2.44%), mast cells (0.99%), endothelial cells (0.97%), erythrocyte (0.58%), and dendritic cells (DCs, 0.51%) (**Figures 1B**, **S1D**). Furthermore, the clusters displayed in t-SNE were labeled according to sample source and disease state (**Figures 1C, D**; **Figures S1E, F**). Furthermore, those clusters were assigned to known cell lines through marker genes (**Figure 1E**, **Figures S1G, H**). Compared with healthy control spleen tissue, there was a deficiency of neutrophils in HS, an increase in dendritic cells, erythrocyte, HSC, mast cells and NK cells (**Figures S1E, F**).

In HS samples, T-cells and B-cells were the main cell subsets (accounting for 86.8%), and significant differences in T-cells and B-cells were found among different types of HS (**Figure 1F**). T-ells accounted for the highest proportion in PH037, and other HS samples had the same proportion of T-cells as the control sample (**Figure S1D**). The proportion of B-cells in PH027 was similar to that of the control sample, followed by PH024 and PH026 samples, while the lowest proportion was found in PH037 at number less than half that of the control. For NK cells, only PH027 was lower than the control sample, and other HS samples contained more than twice that of control sample. The number of HSC in PH037 was three times that of the control, while in PH027 it was only half that of the control. Other cell types with lower proportion also showed great differences among normal spleen and HS, such as dendritic cells (DCs) and mast cells (extremely rare in PH027) (**Figure 1F**, **Figure S1D**). Overall, T-cells and B-cells are the core cells in the spleen, and the broad changes of transcriptional profiles and cell proportions in HS were characterized, which may be the main cause of immune disorder.

### T-Cell Clustering and Subgroup Analysis

**Figure 2:**
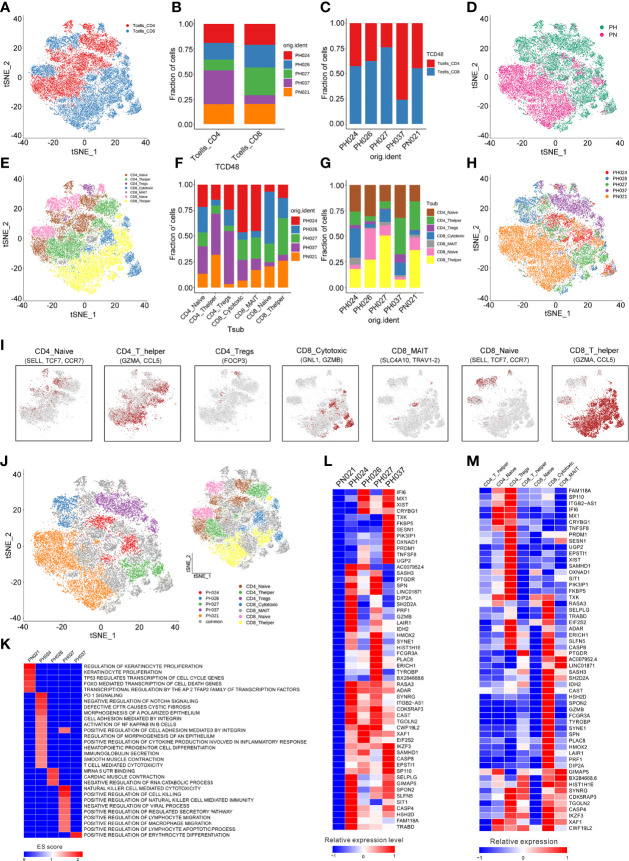
Identification of T-cell clusters. **(A)** The cell distribution of CD4^+^ T-cells and CD8^+^ T-cells. **(B)** The proportions of CD4^+^ T-cells and CD8^+^ T-cells in different splenic samples. **(C)** The proportions of CD4^+^ T-cells and CD8^+^ T-cells in each splenic sample. **(D)** t-SNE plot of the B-cell distribution in health control and HS group. **(E)** The t-SNE plot revealing seven T cell clusters. **(F)** Relative proportion of each HS across different T-cell clusters as indicated. **(G)** Relative proportion of each T-cell subcluster in five splenic samples as indicated. **(H)** t-SNE plot of T-cells from 5 splenic samples (indicated by color). **(I)** t-SNE plot of T-cell subclusters, color coding for the expression of the marker genes (gray to red) for the indicated cell subtype. **(J)** t-SNE plot showed specific T-cells from different splenic samples. **(K)** GSEA enrichment of T-cells in different splenic samples. **(L)** The heatmap of the expression of T-cells specific genes on each splenic sample. **(M)** The heatmap of the expression of T-cells specific genes on each cell subcluster.

Next, a total of 26,528 T-cells were detected in all samples, and were classified to 29 clusters through t-SNE (**Figures S2A–C**). According to the CD4 and CD8 makers, 12 clusters belonged to CD4^+^ T-cells (44.33%), and 17 clusters belonged to CD8^+^ T-cells (55.67%) (**Figure 2A**). Among all the HS, more CD4^+^ cells than CD8^+^ cells were found in PH307, while in PH027, CD8^+^ cells were more numerous than CD4^+^ cells; Other samples were the same as that in the healthy control (**Figures 2B–D**). In comparison, HBV-infected spleens had more CD4^+^ T-cells; HCV-infected spleens had more CD8^+^ T-cells (**Figures 2B–D**). Then, T-cells were further marked by other genes and clustered to seven groups, including CD4^+^ Naive cells (21.56%, *SELL*), CD4^+^ T helper cells (20.39%, *GZMA*), CD4^+^ Tregs cells (2.38%, *FOCP3*), CD8^+^ T helper cells (31.06%, *SELL*), CD8^+^ Naïve cells (12.21%, *SELL*), CD8^+^ Cytotoxic cells (9.56%, *GNL1*), CD8^+^ MAIT cells (2.84%, *SLC4A10*) (**Figures 2E, I**; **Figure S2D**). Of these seven T-cell subpopulations, CD4^+^ Naïve cell, CD4^+^ T helper cell, and CD8^+^ T helper cell groups were the core cell groups, accounting for 73.01% of all T-cells, and they showed different abundances in different HS samples (**Figures 2F–H**). Only the number of CD4^+^ Naïve cells of PH027 was equivalent to that of the control, and that of other HS samples were about twice as high as the control. The content of CD4^+^ T helper cells in PH037 was higher than that in the control sample, while that in other HS samples was lower than in the control, and it was the lowest in PH026. The content of CD8^+^ T helper cells in PH027 sample was higher than that in control sample, while it in other HS samples was lower than control, and it was the lowest in PH037 (**Figures 2F, G**). T-cells were labeled according to the source of the sample, and HS and normal spleen were roughly separated using t-SNE (**Figure 2H**).

To study T-cell subclusters in different HS types, a functional enrichment analysis was conducted on the enriched genes and the specific marker genes for each T-cell subclusters were screened out. Among these T-cells, some subclusters were specifically present in the sample. For example, one population of CD4^+^ Naïve cells and one population of CD8^+^ Cytotoxic cells were specifically expressed in PH024 (**Figure 2J**). The GSEA enrichment results, which were analyzed based on the enriched genes of specific expressed T-cell subclusters, indicated that the specific T cell in PH037 had stronger red blood cell differentiation signals, and the specific T-cell subclusters in PH024 sample had stronger PD-1 signals (25, 26), Cystic Fibrosis (27), and Polarized epithelium signals (28), and the specific T-cells in PH027 sample had stronger positive regulation signals for natural killer cell-mediated immunity. PH024 and PH027 were simultaneously enriched in cell adhesion mediated by integrin (**Figure 2K**). Among those high-expressed genes in T-cells, we found none were highly expressed in the healthy sample (**Figure 2L**). However, there were 55 highly-expressed genes in HS, and half of them were highly expressed in all HS samples. For instance, *RASA3*, *ADAR*, *XAF1*, and *IKZF1* are generally highly expressed in T-cells in HS and are associated with immunosuppression (**Figure 2L**). In particular, *ADAR* was found to be a target for cancer immunotherapy (29). Furthermore, there were some genes that were only highly expressed in one HS. *TNFSF8* was highly expressed in PH037 alone and was also enriched in CD4^+^ Naïve T-cells and CD4^+^ Treg cells (**Figures 2L, M**). Furthermore, previous research have found that *TNFSF8* was associated with regulation of immune response (30) and high risk of hepatitis infection (31). In general, our research found that abnormal distribution and proportion of T-cells can be a characteristic of HS; these T-cell enriched functions and genes have certain characteristics, which are mainly manifested through immunosuppression.

### B-Cell Clustering and Subgroup Analysis

**Figure 3:**
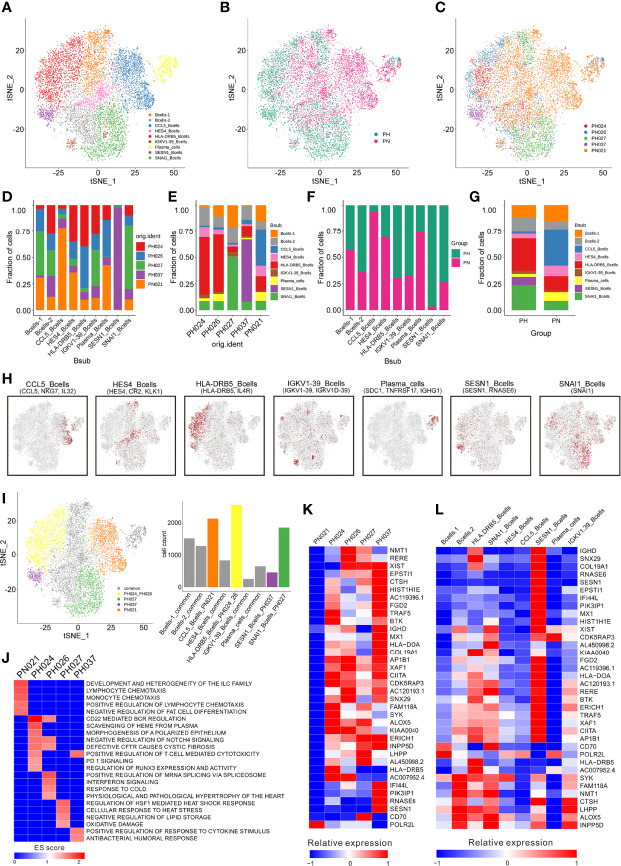
Identification of B-cell clusters. **(A)** t-SNE plotting revealing nine B-cell clusters. **(B)** t-SNE plot of the B-cell distribution in the normal control and HS group. **(C)** t-SNE plot of B cell subtype in different samples. **(D)** Relative proportion of each HS across different B-cell clusters as indicated. **(E)** Relative proportion of each B-cell subcluster in five samples as indicated. **(F)** Relative proportions of healthy controls and HS in different B cell clusters. **(G)** Relative proportion of the B-cell subclusters in the normal control and HS group. **(H)** t-SNE plot of B-cell cluster, color coding for the expression of the marker genes (gray to red) for the indicated cell subtype. **(I)** t-SNE plot showing specific B-cells from different splenic samples. **(J)** GSEA enrichment of B-cells in different splenic samples. **(K)** The heatmap of the expression of B-cell specific genes on each splenic sample. **(L)** The heatmap of the expression of B-cells specific genes on each cell subcluster.

A total of 11,500 B-cells were classified into 13 clusters (**Figures S3A–C**), and these cells were further categorized into nine subclusters according to marker genes (**Figures 3A–C, H**). Firstly, HS was found to be significantly deficient in *CCL5* ^+^ B-cells, while many *SESN1* ^+^ B-cells and *SNAI1* ^+^ B-cells are enriched (**Figures 3B, F, G**). In addition, the distribution of B cell subclusters in different HS varies. Among B-cell subclusters, *CCL5* ^+^ B-cells were found to be specifically present only in healthy control, while *SNAI1* ^+^ B cells in PH027, and *SESN1* ^+^ B cells in PH037. And the *HLA-DRB5* ^+^ B-cells were highly expressed in PH026 and PH027 (**Figures 3C–E**).

The GSEA enrichment analysis were performed based on the enriched genes in specific expressed B cell subclusters and showed that the specific B-cell subclusters in the healthy sample had stronger cell chemotaxis signals (**Figures 3I, J**). The specific B-cell subclusters in PH024 had stronger PD-1 (25) and polarized epithelium signals (28). The specific B-cell subclusters in PH026 had stronger mRNA splicing regulatory signals, and the specific B-cell subclusters in PH027 had stronger heat shock response signals, and the specific B-cell subclusters in PH037 had stronger cytokine responses and humoral response signals (**Figure 3J**).

Among those highly expressed genes screened in B cells, only *POLR2L* was highly expressed in the healthy sample (**Figure 3K**), which was reported as a part of the core element for RNA polymerases (32). Among the B cells, *POLR2L* was mainly expressed in B cell-1, *CCL5* ^+^ B cells and plasma cells (**Figures 3K, I**). There were 35 genes that were only highly expressed in HS samples, and most of them were highly expressed in more than one HS samples, but some genes were only highly expressed in one HS sample. *BTK*, *SYK*, and *HLA-DRB5* were only highly expressed in PH024, and *BTK* is a small-molecule inhibitor which exhibited impressive anti-tumor activity in clinical studies in patients with various B-cell malignancies (33). *NMT1* and *RERE* were only highly expressed in PH026, and *NMT1* was studied widely for breast cancer and showed suppression on initiation, proliferation and invasion of breast cancer cells (34). *CD70* was only highly expressed in PH027, and it was implicated in tumor cell and regulatory T-cell survival through interaction with its ligand (35). *PIK3IP1*, *RNASE6*, and *SESN1* were only highly expressed in PH037, and those genes also highly expressed in *SESN1* ^+^ B-cells (**Figures 3K, L**). While *SESN1* has been studied and found to be associated with p53 mutations (36). Collectively, the abnormality of B cells could be more clearly observed in HS; furthermore, we found the lack of *CCL5* ^+^ B cells and the abnormal presence of *HLA-DRB5* ^+^, *SESN1* ^+^ and *SNAI1* ^+^ B cells in HS were related to the etiology of HS.

### Evolutionary Trajectories of T-Cell and B-Cell Subclusters Among HS

**Figure 4:**
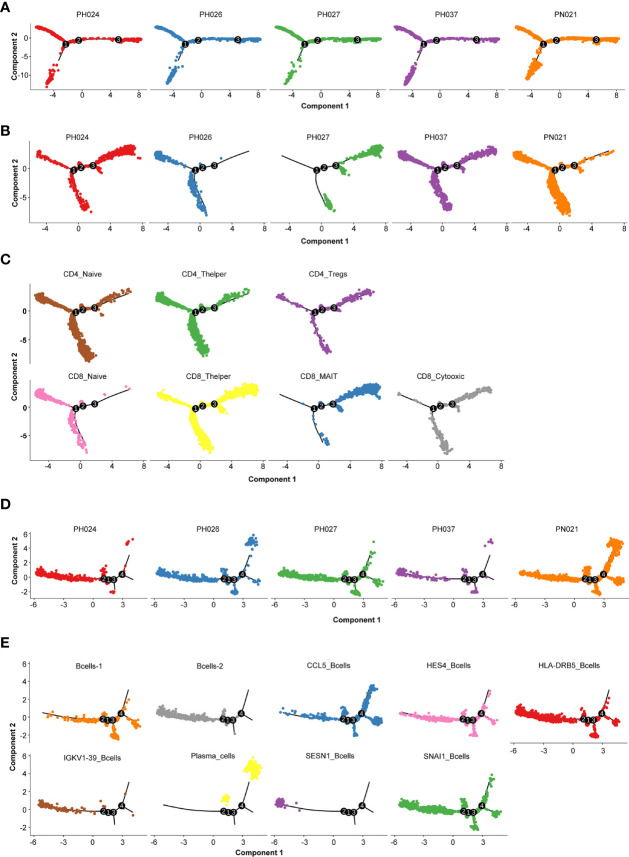
Cell evolution trajectory. **(A)** Evolution trajectory of all cells in different samples. **(B)** Evolution trajectory of T-cells in different samples. **(C)** Evolution trajectory of each T-cell subcluster. **(D)** Evolution trajectory of B-cells in different samples. **(E)** Evolution trajectory of each B-cell subcluster.

To detect the evolutionary trajectory of splenic cells, Monocle 2 was used to perform a pseudo-time analysis for all cells, T-cells, and B-cells, respectively. The results of cell trajectory analysis on all cells showed that no difference existed among different HS samples (**Figure 4A**). HSC and endothelial cells were seen to be precursors to other cells (**Figure S4A**). The pseudo-time analysis based on T-cells and B-cells demonstrated different clear evolutionary trajectories. In the evolution trajectory of T-cells, these cells were distributed on the trajectory chart composed of three branches (progressing from Branch 1 to Branch 3, **Figure 4B**). The developmental trajectories of PH024 and PH037 are relatively consistent. Compared with the control PN021, PH026 T-cells were overly naïve, and PH027 T-cells were senescent (**Figure 4B**). The composition of the corresponding cell subsets developed from CD4^+^ Naïve and CD8^+^ Naïve to CD4^+^ T helper and CD8^+^ T helper, and finally to CD8^+^ MAIT and CD8^+^ cytotoxic cells (**Figure 4C**). All B-cells were distributed on a trajectory diagram composed of four branch points, starting from Branch 2 to Branch 4. B-cells from healthy spleen were evenly distributed on the developmental trajectory, while the trajectory of B cells in HS showed a lack of development (**Figure 4D**). Analysis of the developmental trajectory of B-cell subgroups shows that Bcell-2, *IGKV1-39* ^+^ B-cells, and *SESN1* ^+^ B-cells were in the early developmental trajectory, while *CCL5* ^+^_B-cells and Plasma_B-cell were in a mature functional state (**Figure 4E**). In summary, the developmental trajectory of all cells in HS was not different from that of the control spleen; but the T-cells and B-cells in HS were in a state of incomplete development.

### Significant Differences in Cell-Cell Interactions in HS

**Figure 5:**
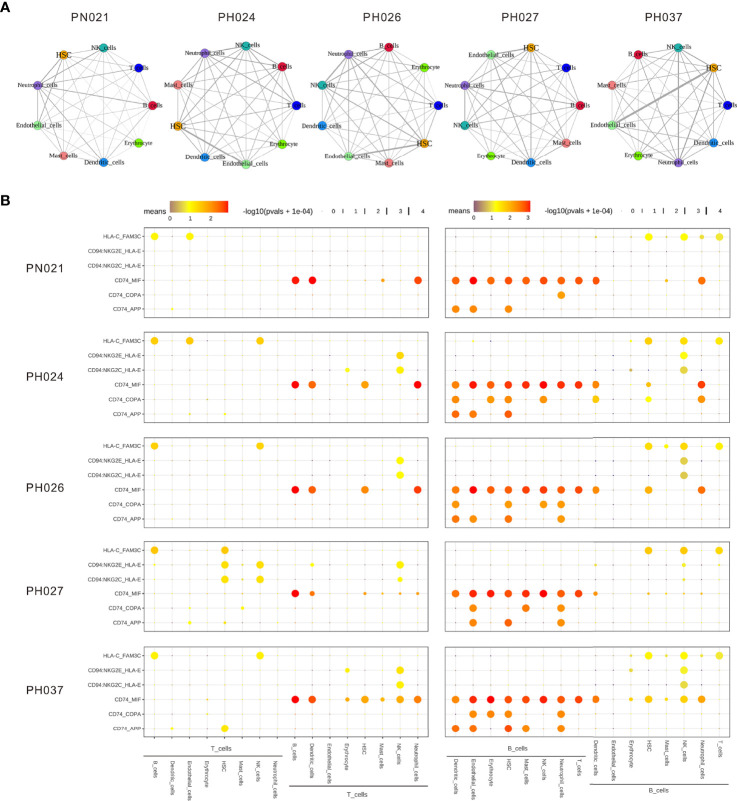
The dense network and multiple regulatory immune responses in each splenic sample. **(A)** Overview of selected ligand-receptor interactions of nine cell subtypes. **(B)** Overview of selected ligand-receptor interactions of T-cells, B-cells, and other cell types.

Next, CellPhoneDB (37) was used to determine the ligand-receptor interactions among HS cells, showing that there were significant differences in ligand-receptor interactions between HS and normal control. The cell group having strongest interaction with the other cell groups involved neutrophil cells in normal spleen, but the HSC and endothelial cells were the strongest interaction cell groups in HS, especially in PH037 (**Figure 5A**). This finding was consistent with the phenomenon observed by Wang and Katztong such that endothelial cells directly contact HSCs (38). For the ligand-receptor analysis, there were differences among HS and normal spleen (**Figure 5B**). T cells were mainly regulated by B-cells, DCs, and neutrophil cells through CD74-MIF. Compared with the normal spleen, NK cells exerted a negative regulatory effect on T-cells through CD94-HLA-E in HS samples (**Figure 5B**). B-cells in the spleen play a major regulatory role, whether in HS or control. B-cells mainly interacted with other cells through CD74. In addition, B cells in HS exert regulatory effects on a variety of cells through CD74-COPA. Importantly, it was found that both T cells and B cells in PH were negatively regulated by NK cells through CD94-HLA-E (39) (**Figure 5B**). In summary, abnormal enhancement of the interaction between epithelial cells and HSC in HS, and T/B-cells in HS was found to play a regulatory role through CD94-HLA-E and NK cells.

### Specific Maker Genes of Other Cell Types in HS

**Figure 6:**
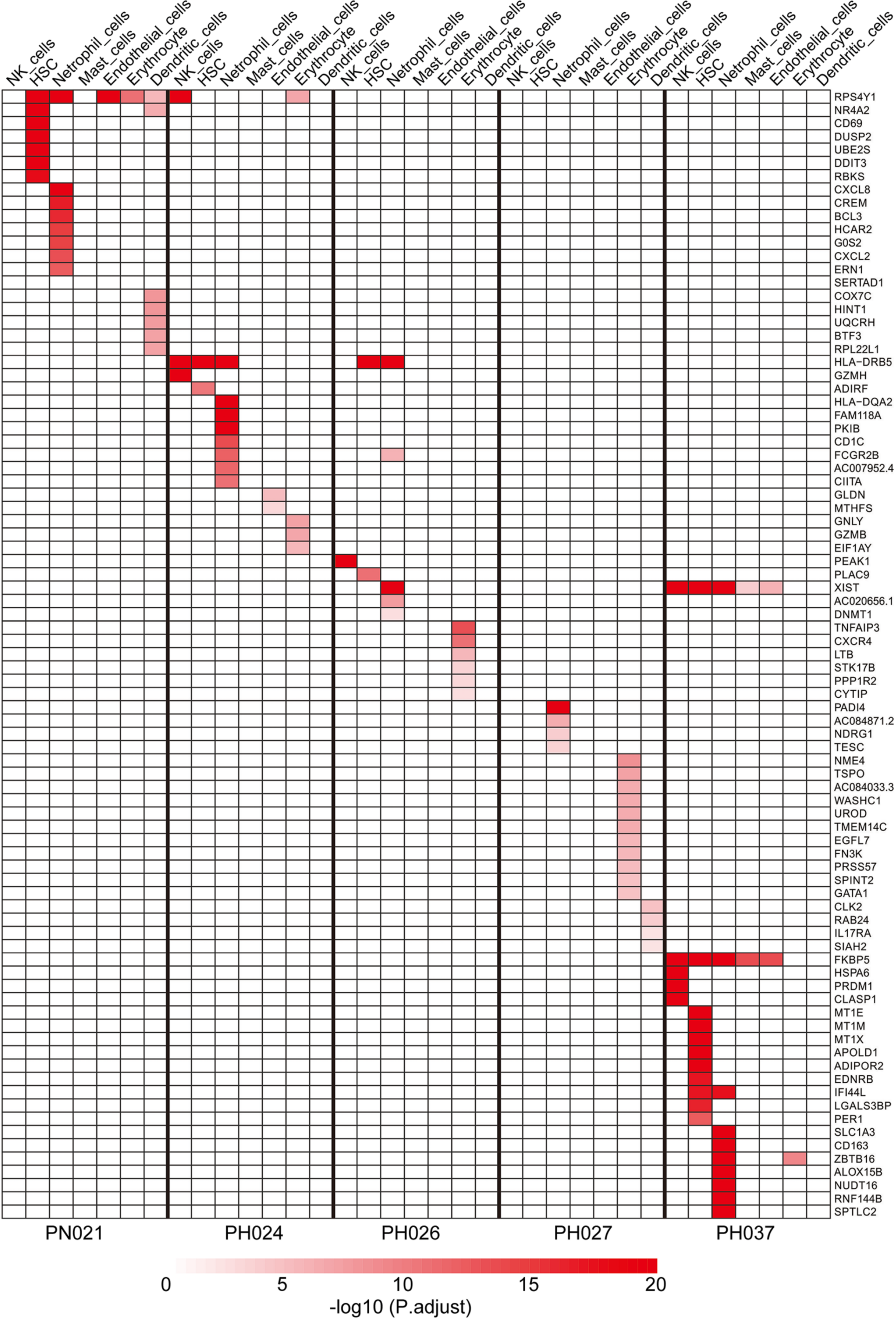
Specific marker genes for different HS samples in other cell clusters.

**Figure 7:**
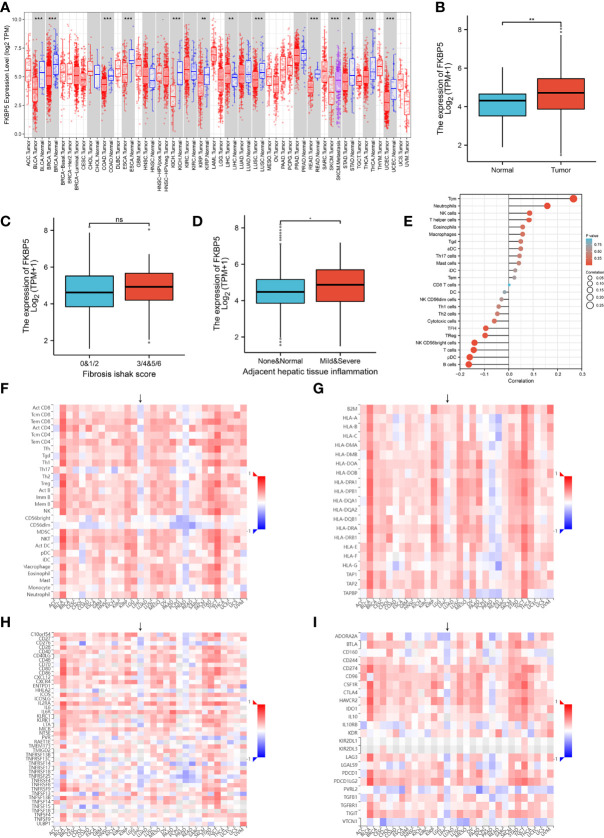
Verification using TCGA data for FKBP5. **(A)** Pan-cancer expression of *KFBP5*. **(B)** Box plot showing the expression of *KFBP5* mRNA in primary HCC tissues and normal tissues. **(C)** Box plot showed the association between KFBP5 expression and fibrosis Ishak score. **(D)** Box plot reflecting the association between KFBP5 expression and tissue inflammation. **(E)** Correlations between the relative abundance of 24 immune cells and expression level of *KFBP5*. The size of the dots represents the absolute Spearman’s correlation coefficient values. **(F)** Heatmap showing Spearman correlations between Expression (exp) of FKBP5 and lymphocytes (Y-axis) across human cancers (X-axis). **(G)** Heatmap showing Spearman correlations between expression (exp) of *FKBP5* and MHC molecules (*Y*-axis) across human cancers (*X*-axis). **(H)** Heatmap showing Spearman correlations between expression (exp) of *FKBP5* and immune-stimulators (Y-axis) across human cancers (X-axis). **(I)** Heatmap showing Spearman correlations between Expression (exp) of *FKBP5* and immune-inhibitors (*Y*-axis) across human cancers (*X*-axis). ***P< 0.001, **P< 0.01, *P< 0.05, ns, no significance.

Except for T-cells and B-cells, other cells accounted for 13% of the total cell count, but there were also specific marker genes enriched in these clusters. A total of 184 specific marker genes were screened from these cells. *RPS4Y1* was highly-expressed in a variety of cells in the normal spleen (**Figure 6**). Through database analysis, we found *RPS4Y1*, a member of the S4E family of ribosomal proteins (40), was poorly expressed in a variety of tumors including LIHC (**Figures S5A, B**). The high expression of RPS4Y1 was associated with high fibrosis and high immune infiltration in the liver (**Figure S5C, D**), and the expression of *RPS4Y1* was positively correlated with Tcm, Tem and pDC (**Figure S5F**). High expression of *PRS4Y1* indicated a better prognosis (**Figure S5E**). Furthermore, FKBP5 was found to be specifically expressed in a variety of cells in PH037 (**Figure 6**), and it was also enriched in Treg cells in HS (**Figure 2M**). Previous research indicated that *FKBP5* was related to immune response, and the high expression of *FKBP5* was linked to a pro-inflammatory profile and altered NF-κB-related gene networks (41). In our study, *FKBP5* is highly expressed in a variety of tumor tissues (**Figure 7A**). In liver cancer, *FKBP5* was related to tissue type and immune infiltration, but not to the degree of fibrosis (**Figures 7B–D**). *FKBP5* is negatively correlated with B cells, DCs and T cells in the microenvironment (**Figure 7E**). In the analysis of immune cells and immune factors, FKBP5 specifically and negatively correlated with a variety of immune effector cells and immune effector factors in the microenvironment of liver cancer (**Figures 7F–I**). In addition, *HLA-DRB5* was enriched in HSC and neutrophils of PH024 and PH026; *XIST* was enriched and expressed in PH026 and PH037. Collectively, only a small number of genes in HS were common in different HS samples, while the expression of *RPS4Y1* is generally absent in HS, and *FKBP5* is enriched and expressed in HCC-HS.

## Discussion

As an important organ of the human body, the spleen can not only filter blood but also play a role in the immune system (42). HS is a common splenic disease which is characterized by its overactivity and blood cell clearance (1). Splenectomy is the most important treatment for HS with significantly relieved symptoms at the post-operative stage (3), and splenic embolization is usually used for patients in poor health (43). Many patients with HCC may also suffer from other liver diseases, such as cirrhosis, infection by HBV or other viruses (44). Some HCC patients with liver diseases usually also have thrombocytopenia caused by HS (6, 8, 43). The latest meta-analysis found that splenectomy can improve the prognosis of patients with liver cancer (45), but the specific mechanism of action is unknown. A deeper understanding of the mechanism of HS is an important basis for better HS treatment. Most previous studies only focused on one type of HS. In our study, four different types of HS and a healthy spleen were selected. To obtain high quality data, samples were collected according to standard and low-quality cells were filtered by sequencing.

Compared with previous studies, we provided an in-depth description of the composition of cell populations in HS. T-cells and B-cells were the core cell types in all samples, indicating the important immune functions of spleen. The subtype identification for the core cell types showed that CD4^+^ naïve cells, CD4^+^ T helper cells, and CD8^+^ T helper cells are the core cell subtypes in all T-cells. The vast majority of T-cells in HS present a naïve or immunosuppressive state. Furthermore, the ratio of CD4^+^/CD8^+^ in the spleen with HCC is imbalanced, and more CD4^+^ Treg cells can be captured, which may be the reason for the accumulation of Treg in the HCC microenvironment (46).

The functions and numbers of these significantly expressed genes were different in each sample, implying the genetic difference for each HS. Most of enriched genes were reported for the first time to be enriched in HS samples, due to the limitation of previous studies on the transcriptome of HS-PHT and single gene knock-outs. In the previous study, *PIK3R1* was reported to be up-regulated significantly in macrophages of HS-PHT, and the knock-down experiment showed the inhibition of its expression might be useful for HS-PHT treatment (47). In present study, the specific expression of *PIK3R1* was detected in both T-cells and B-cells of the PH037(HS-HCC) sample but was not enriched in PH026 (HS-PHT) samples. We also enriched some genes related to immune and diseases, such as the specific expression of *TNFSF8* in T-cells of HS samples, *FKBP5* specific expressed in T cells and other cells of HS-HCV. In addition, some enriched genes in T cells were also found in HS. *DIP2A* and *SH2D2A* and both were highly expressed in the CD8^+^ cytotoxic cells. In addition, *DIP2A* was associated with abnormal brain development and brain diseases (48), and *SH2D2A* was important for proper activation of T-cells (49). Only *TYROBP* and *BX284668.6* were highly expressed in PH027, and only *TYROBP* was highly expressed in CD8^+^ cytotoxic T-cells, while *BX284668.6* was highly expressed in multi CD8^+^ T-cells. *TYROBP* was reported as a potential prognostic biomarker of clear cell renal cell carcinoma and was highly related with Alzheimer’s disease (50, 51). There were also some genes specifically high-expressed in PH037, including *TXK*, *FKBP5*, *SESN1*, *PIK3IP1*, and *OXNAD1*. *TXK*, which specifically regulates interferon-gamma gene transcription. *FKBP5*, highly expressed in CD4^+^ Tregs of PH037, was related to immune response, and its high expression was linked to a pro-inflammatory profile and NF-κB pathway (41, 52, 53). *SESN1* as a novel molecular target can improve head and neck cancer treatment (54). *PIK3IP1* was reported as a negative regulator for T-cell immunity (55), and *OXNAD1* mutation was reported to be linked to skin cancer (56). Furthermore, these genes were highly expressed in CD4^+^ Tregs.

In the study of B-cells, we found that compared with healthy spleens, HS lacks *CCL5* ^+^ B-cells. CCL5 is a chemokine that plays an important role in immune regulation. Furthermore, some genes worth studying in B-cells were also detected. In addition, *RNASE6* showed antibacterial activity in previous trials (57). Previous studies have found that SESN1 mediates the inhibition of cell growth by p53 by activating AMP-activated protein kinases (36). Compared with the normal control, these enriched genes may play important roles in the pathogenesis of HS, warranting validation in the future.

In addition to T/B-cells, we also performed a gene enrichment analysis for other cells to strengthen our understanding of the mechanism of HS. *CD69* was highly enriched in the HSC of the control sample and associated with eosinophilic pneumonia, and this protein may act to transmit signals in natural killer cells and platelets. *BCL3* was enriched in neutrophil cells of control sample, and contributes to the regulation of cell proliferation (58). *GZMH* was enriched in NK cells of HS-HBV sample, that plays a role in the cytotoxic arm of the innate immune response by inducing target cell death and by directly cleaving substrates in pathogen-infected cells (59). *PEAK1* was specifically expressed in NK cells of HS-PHT sample, that plays a role in the regulation of cell migration, proliferation and cancer metastasis (60). *DNMT1* was enriched in HS-HCV, which associated at promoter regions of tumor suppressor genes (TSGs) leading to their gene silencing (61). *TNFAIP3* was enriched in HS-HCV, which was reported to be involved in immune and inflammatory responses signaled by cytokines and has a role in the function of the lymphoid system (60).

In the pseudo-time analysis based on marked T-cells and B-cells, HS-HCC and HS-HBV were all closely connected, but the two evolution trajectories showed different positions of HS-HCV compared to that in the normal control(this should be further discussed based on a greater number of samples). The T/B-cells enriched in HS exhibited abnormalities in the developmental trajectory, among which PH037 was the most obvious.

Subsequently, the interactions of various cells in HS were elucidated through cell interaction and ligand-receptor analyses. The cell-cell interaction analysis found that the main interaction cells in HS are epithelial cells and HSC. Peripheral blood cell reduction is one of the most common complications of HS and the spleen can regulate the function of HSC by regulating EGF signals through epithelial cells (62). However, Lu et al. (38)found that epithelial cells can directly contact HSC to function through the MERFISH spatial transcriptome. Ligand-receptor analysis showed that T cells and other cells in the spleen were regulated by B-cells *via* CD74. In addition, NK cells simultaneously play a regulatory role on T-cells and B-cells through CD94-HLA-E. Previous studies have found that NK cells activate T/B-cells earlier and exert a regulatory effect thereon (63). Combining the characteristics of the distribution of B cell subpopulations, it is inferred that there is a lack of *CCL5* ^+^ B-cells in HS, and there are multiple immunosuppressive B-cell subpopulations (*HLA-DRB5* ^+^ B-cells, *SESN1* ^+^ B-cells, and *SNAI1* ^+^ B-cells). These abnormal B-cell subclusters regulate a variety of cells (including T cells) through CD74, which in turn leads to a systemic immune imbalance. After splenectomy, abnormal B-cells are removed, and normal immunity is restored. Cell communication analysis also provides potential drug targets for treating the disease.

In summary, we have innovatively drawn a whole-cell map of HS. These results implied that HS is an immune disorder with a variety of cell imbalances caused by B cell (mainly *CCL5* ^+^ B cell deficiency). Many immune-related genes were specifically expressed in HS, especially *FKBP5* and *RPS4Y1*. The significantly enriched genes and pathways provide new insights into the genetic and molecular characteristics of HS, which is critical to develop more potential treatments for HS.

## Materials and Methods

### Patients

In this study, four patients with HS (group: PH) and a patient with normal spleen (group: PN) were selected. The splenomegaly of PH group patients was caused by different reasons, including hepatitis B virus infection (HS-HBV, PH024), portal hypertension (HS-PHT, PH026), hepatitis C virus infection (HS-HCV, PH027), and hepatocellular carcinoma (HS-HCC, PH037). The patient with a normal spleen was used as control (PN027), whose spleen was ruptured due to trauma.

This study was approved by the Ethics Committee of Shanxi Bethune Hospital, and complied with all relevant ethical regulations (SBQLL-2020-038). Each participants signed an informed consent proforma.

### Sample Collection

Samples were freshly collected from patients during surgical resection. Firstly, the spleen in the abdominal cavity was removed from its blood supply and quickly excised by surgical instruments. To maintain cell viability, each specimen was immediately immersed in culture medium and stored in separate 50 ml centrifuge tubes with 10% fetal bovine serum (FBS). Then, about 0.1-1 g spleen tissue was transferred into a 100-mm Petri dish and was cut into 1-2 mm tissue blocks using sterilized scissors and tweezers. The tissue blocks were transferred to a 10 ml centrifuge tube and were washed two times by adding PBS solution, centrifuging at 300g for 5 minutes, and removing the supernatant. Thereafter, the tissue blocks were transferred to cryopreservation tube and 1 ml of cryopreservation fluid was added (CELLBANKERTM2, ZENOAQ). After standing at room temperature for ten minutes, the samples were stored at -80 °C for further investigation. All laboratory work in this study was conducted in a biological safety cabinet to prevent contamination.

### Single-Cell Suspension Preparation

To maintain cell structure, spleen tissue was dissociated into single-cell suspensions by enzymatic degradation of the extracellular matrix. Firstly, the tissue was washed by using ice-cold PBS buffer and soaked in 30 mM EDTA solution for 20 mins on ice to inhibit nuclease activity and reduce the stability of the cell membrane. The tissue was then washed again using PBS buffer, and 2 mg/ml collagenase IV (Worthington Biologicals) was used to dissociate tissue into single cells at 37°C for 20 minutes. To maintain cell viability, the single-cell suspension was put at ice and 10% FBS solution added. After dissociation, the suspension was filtered through a 40-um cell strainer (BD Falcon, BD Biosciences, San Jose, CA, USA) to remove large particles.

### ScRNA-Seq Library Construction and Sequencing

Single-cell barcoding and scRNA-seq library preparation were performed based on 10X Genomics single-cell RNA sequencing platform (10×Genomics, Pleasanton, CA, USA). Before barcoding, the cell concentrations of single-cell suspensions were counted using a hemocytometer (TC20, Bio-Rad, Hercules, CA, USA) and adjusted to 1,000 cells/μl. Single-cell suspensions were loaded on the Chromium Single Cell Controller Instrument (10× Genomics, Pleasanton, CA, USA) to generate single cell gel beads in emulsions (GEMs), and 10×Genomics Chromium barcoding system was adopted to construct 10×barcoded cDNA library according to the manufacturer’s instructions. All libraries were sequenced on the Illumina HiSeq X Ten sequencing platform (Illumina, San Diego, CA, USA) with 150 bp pair-end module.

### Sc-RNA Sequencing Data Pre-Processing

The CellRanger ™ pipeline (v3.0.0) was used to pre-process the raw sequencing data, including demultiplexing of cellular barcodes, evaluation of the total number of cells, and applying map reads to the genome. Briefly, after demultiplex cellular barcodes, the total number of cells captured in each sample were evaluated based on CellRanger ‘cell ranger count’ embedded function. The generated data were then mapped to the GRCh38.p5 human reference genome using the STAR aligner (https://github.com/alexdobin/STAR). Finally, the gene expression matrices of each cell were obtained for downstream visualization. After use of the CellRanger™ pipeline, a filter criterion was used to obtain high-quality cells. To remove multiple captured cells, the unique molecular identifier (UMI) count matrix (64) was calculated using R package Seurat (v2.3.4) (https://satijalab.org/seurat/), and the cells with UMI from 500 to 10,000 were selected. The double T-cells generated from two or more cells, were also removed using ‘DoubletFinder’ in Seurat. For each sample, we filtered the genes detected in fewer than three cells and cells with the total number of detected genes (nGenes) less than 500 and more than 3000. Following visual inspection of the distribution of cells by the fraction of mitochondrial genes expressed, low-quality cells where more than 25% of the counts belonged to mitochondrial genes, were further discarded.

### Characterization of Cell Clusters

The remaining cells and genes were used for cell clustering, and the normalization of gene expression was performed on the filtered matrix using by ‘NormalizeData’ function in Seurat. The total expression of each cell was 10,000. To compare transcriptome profiles along five different samples, we merged five different datasets using ‘merge’ of Seurat (65). The FindVariableFeatures tool was used to identify variable genes whose expression was between 0.125-3 times the average level of gene expression. All variable genes were ranked in descending order and the top 2,000 variable genes selected. The Principal component analysis (PCA) on cells was performed on cells on the basis of the expression of variable genes using ‘RunPCA’ in Seurat. Based on the PCA results, the top 30 principal components were selected for clustering analysis based on t-distributed stochastic neighbor embedding analysis, and the FindCluster tool was used to cluster the cells.

To identify cell subtypes, the cell clusters were annotated with the previously reported maker genes. The specifically expressed genes of each cell subtype were studied using the ‘FindMarkers’ function in Seurat, and the differential expressed genes were screened with the criterion (qvalue: 0.01, N0: 20, N1: 1.5, N2: 30%, N3: 3) and divided into three groups: significantly enriched genes (qvalue < 0.01), strictly enriched genes (qvalue < 0.01), and specific molecular marker genes of all cell groups. To sub-group cell clusters of interest for a more in-depth analysis, the ‘SubsetData’ function in Seurat was used to extract each cluster of interest. The extracted subclusters were also annotated with the previously reported maker genes to provide higher resolution for dissecting cellular heterogeneity among particular types of cells. Based on the significantly enriched genes for all samples, gene enrichment was conducted on the specific subclusters of each sample, and the GO and KEGG Pathways for the enriched gene were enriched using the ‘clusterProfiler’ software package.

### Constructing Single-Cell Pseudo-Time Differentiation Trajectories

Based on the significantly enriched genes for each subcluster, cell differentiation trajectories were analyzed. Monocle (v 2.10.0) software (66) was used to order single cells along pseudo-time axis. The pseudo-time analysis tool Monocle (v 2.10.0) can simulate the dynamic changes of time development using machine learning methods (67). First, the significantly enriched genes were selected and performed spatial dimensionality reduction based on their expression. Then, the minimum spanning tree was constructed to determine the differentiation trajectory of the cell. Finally, the continuous evolution process of cells was revealed, and the position and relationship of different cell types in the evolutionary process were also distributed.

### Cell-Cell Interaction Analysis

Cell-cell interactions between different cell types were predicted based on known ligand–receptor pairs by Cellphone DB v2.1.0 (37). Permutation number for calculating the null distribution of average ligand-receptor pair expression in randomized cell identities was set to 1000. Individual ligand or receptor expression was subject to a threshold based on a cut-off based on the average logarithm of the gene expression distribution for all genes across each cell type. Predicted interaction pairs with a *p*-value below 0.05 and of average log expression exceeding 0.1 were deemed significant. The interaction between cells was visualized using Cytoscape (68).

## Data Availability Statement

The data presented in the study are deposited in the Sequence Read Archive (SRA) repository, accession number PRJNA847197.

## Ethics Statement

The studies involving human participants were reviewed and approved by Ethics Committee of Shanxi Bethune Hospital, and complied with all relevant ethical regulations (SBQLL-2020-038). The patients/participants provided their written informed consent to participate in this study. Written informed consent was not obtained from the individual(s) for the publication of any potentially identifiable images or data included in this article.

## Author Contributions

HC-Z and C-ZC designed and performed experiments, analyzed the data, and wrote the paper. H-QS, X-XW, and LZ performed the experiments and analyzed data. J-FH and H-LZ provided human samples. H-LZ and J-FH initiated and designed the study. J-FH initiated the study, organized, designed, and wrote the paper. All authors contributed to the article and approved the submitted version.

## Funding

This work was supported by the Shanxi Science and Technology Department (Grant Nos. 201903D421026, 201901D1111404, and 201901D1111408), Shanxi Province Human Resources and Social Security Department System (Grant No. 20210001), Research Project Supported by Shanxi Scholarship Council of China (Grant No. 2021-116), and Shanxi ‘136’ Leading Clinical Key Specialty (Grant No. 2019XY002). Shanxi Bethune Hospital provided some help in specimen collection.

## Conflict of Interest

The authors declare that the research was conducted in the absence of any commercial or financial relationships that could be construed as a potential conflict of interest.

## Publisher’s Note

All claims expressed in this article are solely those of the authors and do not necessarily represent those of their affiliated organizations, or those of the publisher, the editors and the reviewers. Any product that may be evaluated in this article, or claim that may be made by its manufacturer, is not guaranteed or endorsed by the publisher.
