# Supplementary material for: Single-cell RNA Sequencing Analysis Reveals New Immune Disorder Complexities in Hypersplenism
Source: Front Immunol. 2022 Jul 5;13:921900. doi: 10.3389/fimmu.2022.921900 (PMC9294158; doi:10.3389/fimmu.2022.921900)
Supplement: Supplementary file 1 [file DataSheet_1.pdf]

Supplementary Material

1 Supplementary Figures.

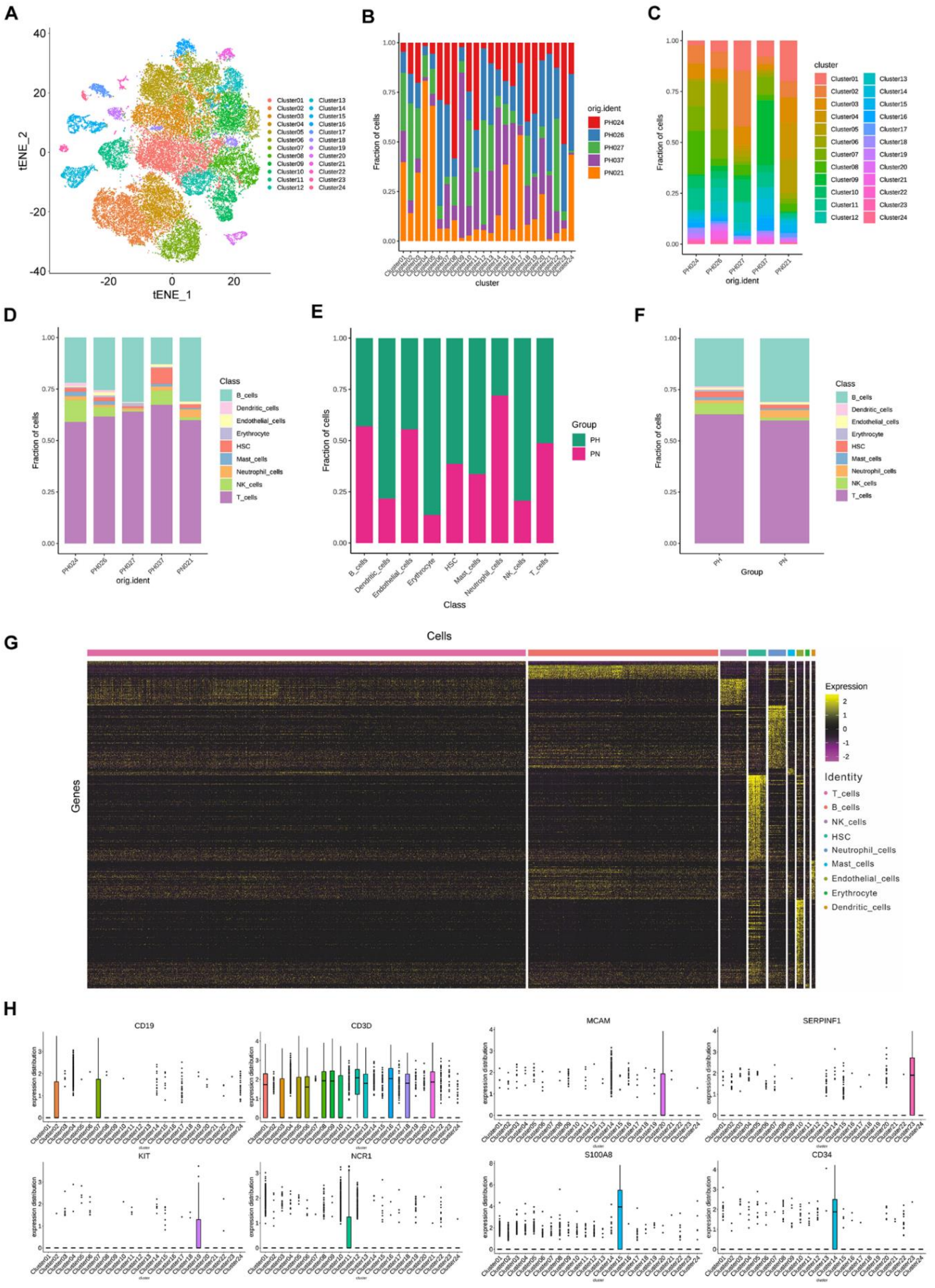
A
tENE_1
tENE_2
B
Fraction of cells
cluster
orig.ident
PH024
PH026
PH027
PH037
PN021
C
Fraction of cells
orig.ident
cluster
D
Fraction of cells
orig.ident
Class
B_cells
Dendritic_cells
Endothelial_cells
Erythrocyte
HSC
Mast_cells
Neutrophil_cells
NK_cells
T_cells
E
Fraction of cells
Class
Group
PH
PN
F
Fraction of cells
Group
Class
G
Cells
Genes
Expression
Identity
T_cells
B_cells
NK_cells
HSC
Neutrophil_cells
Mast_cells
Endothelial_cells
Erythrocyte
Dendritic_cells
H
CD19
CD3D
MCAM
SERPINF1
KIT
NCR1
S100A8
CD34
expression distribution

Supplementary Figure 1. T-SNE plot of cell types, related to Figure 1. **(A)** t-SNE plot of all cells revealing 24 clusters. **(B)** The proportion of different clusters. **(C)** The proportion of 24 clusters in different samples. **(D)** Relative proportion of each cell cluster in 5 splenic samples as indicated. **(E)** Relative proportions of healthy control and HS in different B cell clusters **(F)** Relative proportion of the cell clusters in health control and HS group. **(G)** Heatmap showing expression levels of specific markers in each cell type. **(H)** Expression of marker genes.

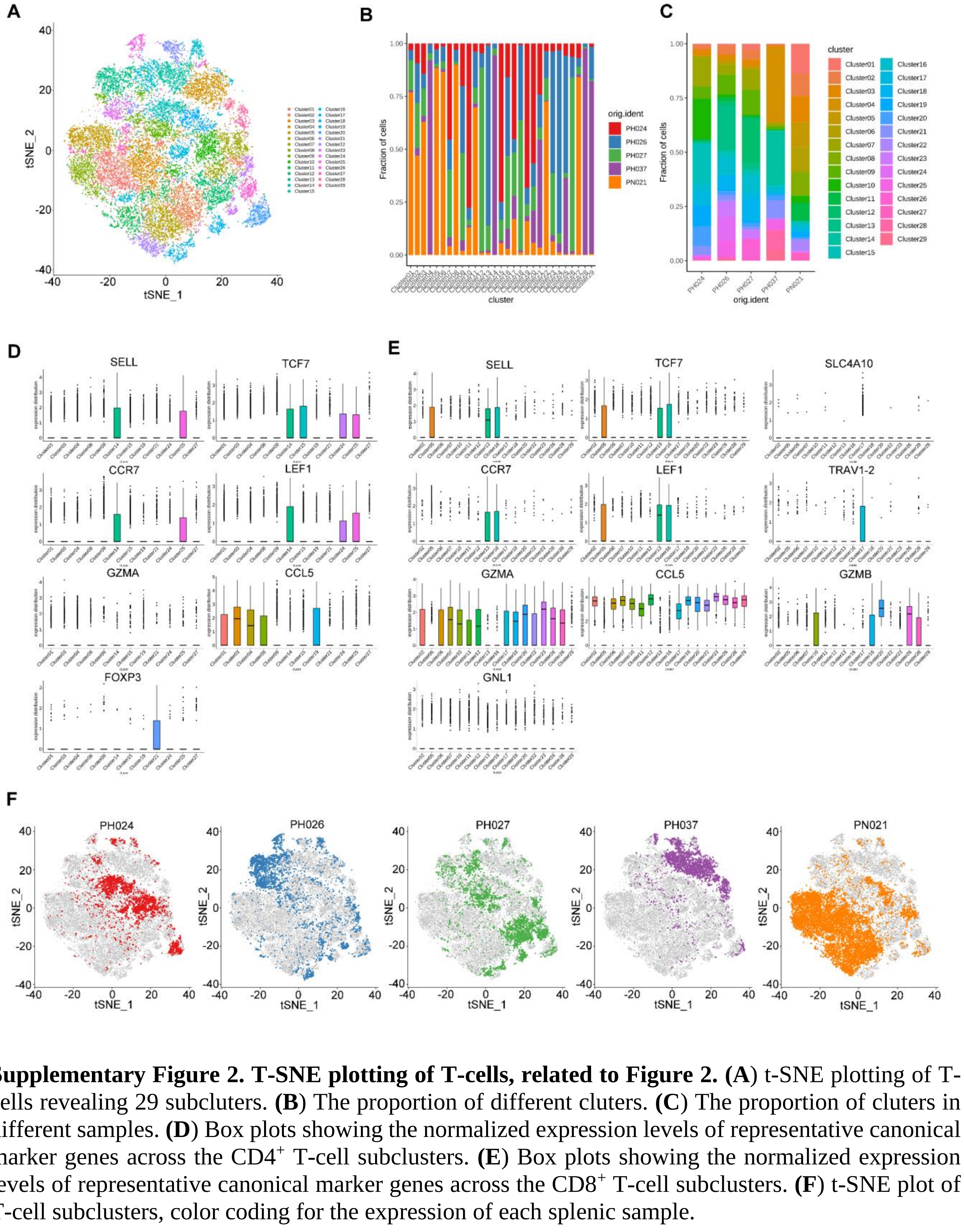

Supplementary Figure 2. T-SNE plotting of T-cells, related to Figure 2. (A) t-SNE plotting of T-cells revealing 29 subcluters. **(B**) The proportion of different cluters. **(C**) The proportion of cluters in different samples. **(D**) Box plots showing the normalized expression levels of representative canonical marker genes across the CD4$^+$ T-cell subclusters. **(E**) Box plots showing the normalized expression levels of representative canonical marker genes across the CD8$^+$ T-cell subclusters. **(F**) t-SNE plot of T-cell subclusters, color coding for the expression of each splenic sample.

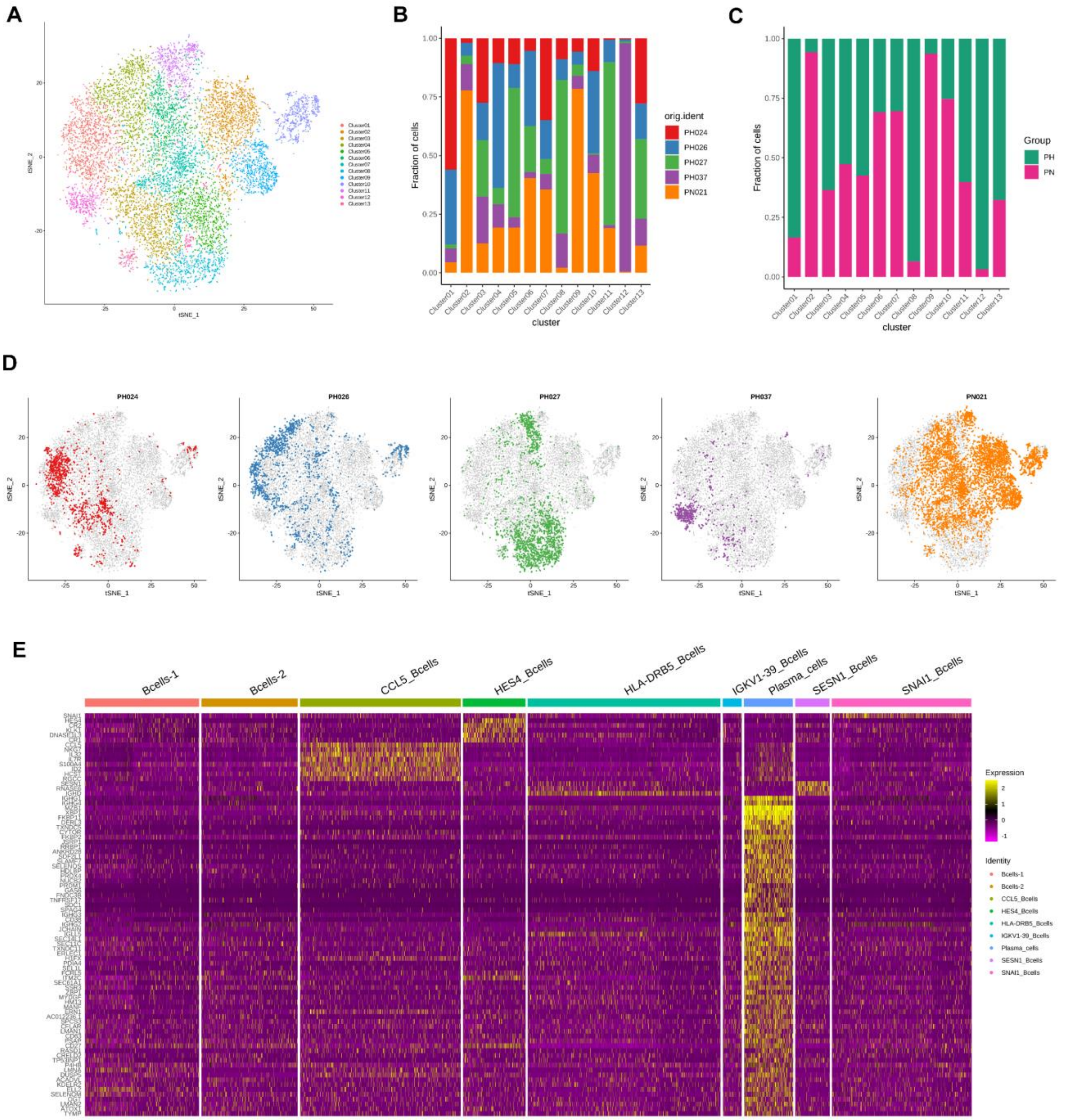

Supplementary Figure 3. tSNE plotting of B-cells, related to Figure 3. (A) tSNE plotting of B-cells revealing 13 subcluters. **(B**) The proportion of different cluters. **(C**) The proportion of cluters in different samples. **(D**) t-SNE plot of B-cell subclusters, color coding for the expression of each splenic sample. **(E**) The heatmap of highly enriched expression in different clusters of B-cells.

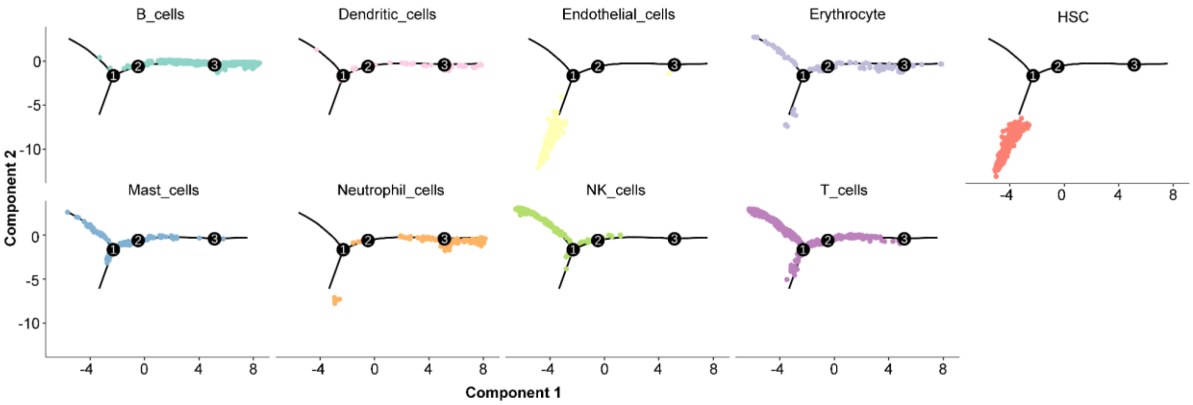

Supplementary Figure 4. Cell evolution trajectory, related to Figure 4. Evolution trajectory of each cell cluster.

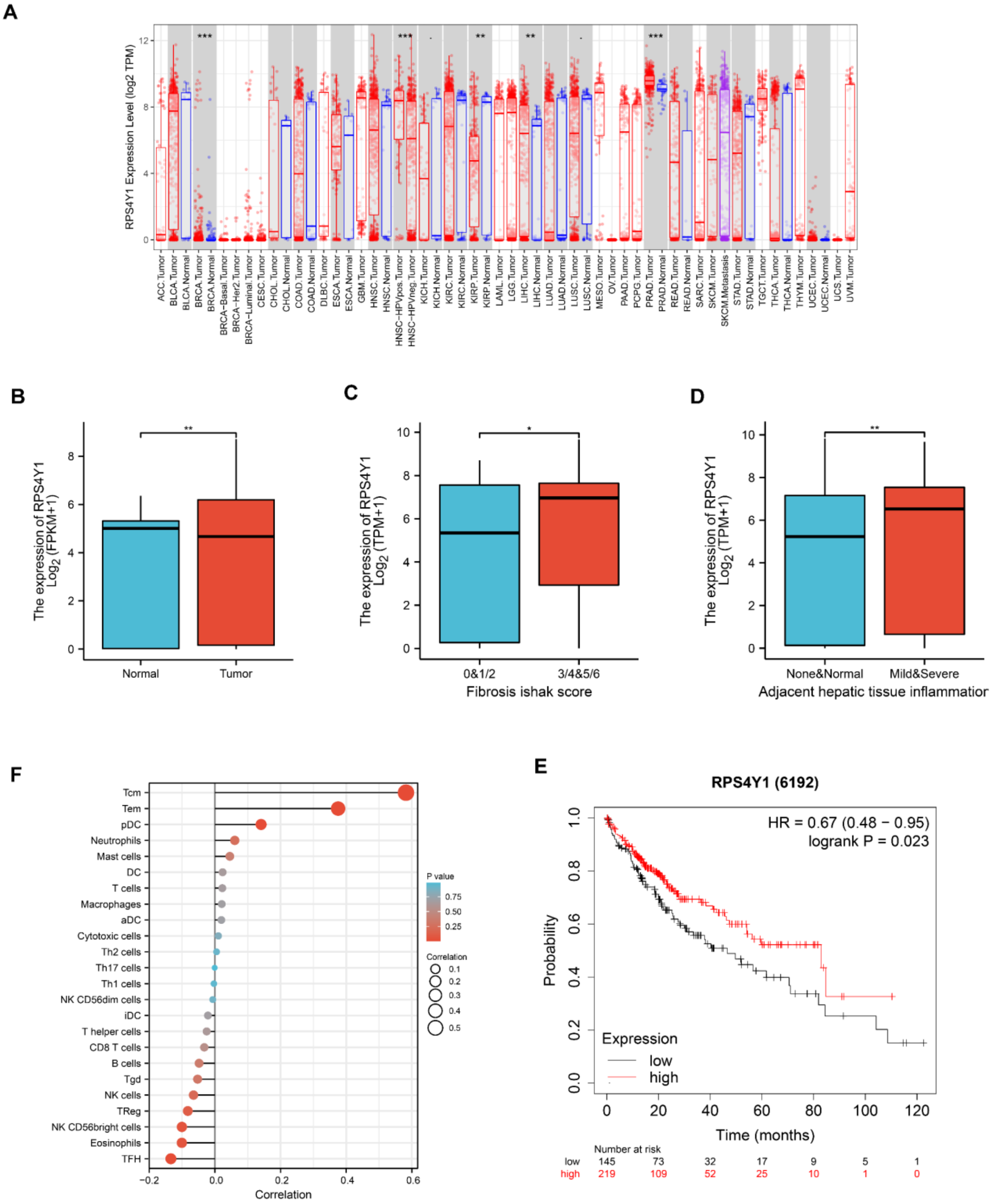

Supplementary Figure 5. Verification using TCGA data for *RPS4Y1*. **(A)** pan-cancer expression of *RPS4Y1*. **(B)** Box plot showed the expression of *RPS4Y1* mRNA in primary HCC tissues and normal tissues. (**C**) Box plot showed the association between *RPS4Y1* expression and fibrosis ishak score. **(D)** Box plot showed the association between *RPS4Y1* expression and tissue inflammation. **(E)** Correlations between the relative abundance of 24 immune cells and *RPS4Y1* expression levels. The size of the dots

represents the absolute Spearman's correlation coefficient values. **(F)** Kaplan-Meier survival curves showed overall survival comparing the high and low expression of *RPS4Y1* in HCC patients.
